# Differential Effects of Physiological Arousal Following Acute Stress on Police Officer Performance in a Simulated Critical Incident

**DOI:** 10.3389/fpsyg.2019.00759

**Published:** 2019-04-09

**Authors:** Eamonn Arble, Ana M. Daugherty, Bengt Arnetz

**Affiliations:** ^1^ Department of Psychology, Eastern Michigan University, Ypsilanti, MI, United States; ^2^ Department of Psychology, Wayne State University, Detroit, MI, United States; ^3^ Department of Psychiatry and Behavioral Neurosciences, Wayne State University, Detroit, MI, United States; ^4^ Institute of Gerontology, Wayne State University, Detroit, MI, United States; ^5^ Department of Family Medicine, College of Human Medicine, Michigan State University, East Lansing, MI, United States

**Keywords:** decision-making, verbal communication, antithrombin, cortisol, stress, police

## Abstract

**Background**: Police officer response in a critical incident is often a life-or-death scenario for the officer, the suspect, and the public. Efficient and accurate decisions are necessary to ensure the safety of all involved. Under these conditions, it is important to understand the effects of physiological arousal in response to acute stress on police officer performance in critical and dangerous incidents. Prior research suggests that physiological arousal following a stressor differentially affects police performance – communication may be impaired, whereas well-rehearsed, tactical behaviors may be resilient.

**Objectives**: In this study, we examine the differential effects of physiological arousal across three police skill domains: verbal communication, nonverbal communication, and tactical skill.

**Methods**: A sample of Swedish police cadets (*N* = 17) participated in a critical incident simulation, which was a reenactment of a real-life incident that had resulted in a police officer death; the simulation included multiple calls, dynamic environments, and surprise threats. An expert rater evaluated the cadets across multiple domains of skill, and physiological arousal was monitored by continuous heart rate monitoring and measures of circulating cortisol and antithrombin taken before and after the incident simulation.

**Results**: The simulation increased police officer arousal, as reflected in elevated heart rate, but this alone did not predict differences in performance. Greater increase in antithrombin was associated with better general performance, but a specific deficit in verbal communication as compared to tactical performance and nonverbal communication. Change in cortisol was unrelated to the skill assessments.

**Conclusions**: Police officer performance during a critical incident simulation is affected by physiological arousal. The findings are discussed with implications for police officer decision-making and real-world performance.

## Introduction

The work of police officers is challenging and dangerous. Police officers are required to operate effectively in the face of physical danger, taxing hours, and institutional burdens. There is a wealth of literature to suggest that the pressures of police work are severe and multifaceted, leading to physical, emotional, and social consequences (Anshel, 2000; Marmar et al., 2006; Reynolds and Wagner, 2007; Arble and Arnetz, 2017). Unfortunately, these negative effects may contribute to the worsening of police officer mental and physical health by disrupting performance during critical incidents (Nieuwenhuys and Oudejans, 2011; Arble et al., 2018). In a critical incident, police officers are sometimes asked to make life and death decisions based upon their assessment of a given situation and the people within them. Mistakes made in these moments bear great consequences for all involved, and the pursuant emotional strain increases the risk of the police officer making mistakes in the future (Klinger, 2006; Leino et al., 2011). The professional necessity of competent law enforcement performance during highly charged situations thus requires a better understanding of the factors responsible for performance within stressful encounters.

“Stress” refers to behavioral and physiological responses that are arousing and aversive, but importantly, whose effects are mediated by cognitive and dispositional factors within the individual (Kim and Diamond, 2002). Following a stressor, there is a complex neurohormonal response that will fluctuate based upon the intensity, nature, and duration of the stressor, as well as several internal factors of the individual experiencing it (Joëls and Baram, 2009). Empirical research distinguishes between acute stress – an ephemeral psychological or physiological response immediately following a stressor – and chronic, or persistent, stress. Although these are convenient theoretical distinctions, their application to understanding the effects of stress on police officer performance is muddled by the nature of police work that presents acute stressors frequently (i.e., multiple incidents a day), as well as sources of chronic stress, such as in anticipation of an incident, shift work, psychological trauma, or negative media coverage (Anderson et al., 2002; Novak et al., 2016). A general theoretical model articulates the effects of stress on skill acquisition and performance follows a nonlinear function: moderate stress and arousal are often adaptive and can bolster performance, whereas high levels of stress and chronic exposure to stress sometimes impair cognitive function and behavioral performance (Yerkes and Dodson, 1908; Sandi, 2013). However, several factors moderate this relationship, including task complexity, and tasks that have multiple or high cognitive demands show a negative relationship with even moderate levels of acute stress (Meunier et al., 2013; Sandi, 2013). Negative effects of acute stress on individual performance of complex tasks are well documented (LeBlanc et al., 2005; LeBlanc, 2009), specifically those assessing memory (de Quervain et al., 2000; Kim and Diamond, 2002), working memory and attentional control (Lupien et al., 1999; Plessow et al., 2012; Schwabe and Wolf, 2012), and decision-making (Cumming and Harris, 2001; Wetzel et al., 2006). Police officer actions during a critical incident stem from these cognitive processes, and therefore, it is useful to consider this theoretical framework when evaluating possible consequences of acute stress on police officer performance.

Several investigations have examined the effects of stress on job performance and decision-making of police officers (Correll et al., 2007; Arnetz et al., 2009; Wright, 2010). Police officers are a useful population for this area of study, because they are often required to make immediate decisions of great consequence across a variety of unpredictable situations. For example, an officer approaching a reportedly armed suspect must attempt to communicate with the suspect while simultaneously visually scanning for the presence of weapons, considering other threats within the environment (e.g., other potential suspects, nearby civilians who could be in danger), evaluating the suspect’s potential escape routes, potentially coordinating movements with a partner, maintaining radio communication, and considering the nature of the suspect in question (e.g., the suspect’s mental state, or if the individual is in fact the actual suspect). These extreme cognitive demands must also be done while the officer is likely to be highly emotionally aroused. In this context of demanding cognitive engagement and emotional arousal, the police officer will be required to make a split-second decision not only to potentially discharge their firearm but also to do so accurately. To add further complication to the matter, many critical incidents arise spontaneously during routine response calls, denying the officers the opportunity to plan or mentally rehearse (Burrows, 2007).

Investigations into the matter have identified numerous factors that contribute to police officer performance during stress. These factors include decision-making styles (Brown and Daus, 2015), dispositional factors (Daus and Brown, 2012), organizational training and culture (e.g., the perception of support from the department; Loyens and Maesschalck, 2010), and situational characteristics (Westmarland, 2005).

Perhaps most critically, acute physiological arousal of police officers has been highlighted as a significant contributor to performance under stress. Acute arousal provides a surge of awareness and energy, increased vigilance to threat, increased responsiveness, and may contribute to successful recovery post-incident (McEwen, 1998, 2006; Munck, 2000; Vonk, 2008; Verhage et al., 2018). In contrast, other functions are found to be impaired during acute arousal, including disrupted visual processing of peripheral information (colloquially referred to as “tunnel vision”; AlSaqr and Dickinson, 2017), declines in executive functioning (McCormick et al., 2007), and difficulty in utilizing environmental feedback (Akinola and Mendes, 2012). Taken together, the cognitive processes and behavioral performance that are required in police work appear to be differentially affected by acute stress, and those functions that characteristically have higher cognitive demands appear to be vulnerable to disruption. One common example of this is the tactical decision to discharge a weapon, and officers under high acute stress conditions or reporting high trait anxiety are more likely to discharge their weapon and have poor shot accuracy (Nieuwenhuys and Oudejans, 2010; Nieuwenhuys et al., 2012). Fatigue, either due to acute or chronic stressors, further alters behavioral choices, including tactical decisions to shoot or pursue a suspect (Hope, 2016). Whereas acute stress or anxiety may impair complex and intentional behaviors, defensive behaviors (Renden et al., 2014) and tactical actions that have been practiced to achieve automaticity (Renden et al., 2017b) appear to be less impaired. Accordingly, intervention studies among police officers have attempted to harness the benefits of physiological arousal, while minimizing its potentially deleterious effects. This intervention research suggests that with extensive practice and repetition, police officers can call upon well-rehearsed tactical procedures under duress, thereby translating physiological arousal into decisive and effective tactical responding (Shipley and Baranski, 2002; Renden et al., 2015, 2017b; Andersen and Gustafsberg, 2016).

Equally important as tactical decisions, an officer ability to effectively communicate is essential to resolving a critical incident safely. Verbal and nonverbal communication are other examples of behaviors that stem from complex and demanding cognitive processing, for which there is evidence of semantic and declarative memory functions to be impaired by acute stress at both very high and very low levels (Sandi and Pinelo-Nava, 2007). Acute physiological arousal (as measured by indices such as cortisol) is predictive of impaired communication skills (Schlotz et al., 2006) with some research suggesting that nonverbal communication (e.g., eye contact) can be predicted by physiological stress levels even prior to the encounter (van Dulmen et al., 2007). In police officers, high acute stress and high trait anxiety predict poor verbal communication during an arrest (Renden et al., 2017a).

Based upon the reviewed evidence, there is seemingly a paradox of police officer performance under stress – aspects of awareness and vigilance are bolstered, whereas behaviors that rely on complex cognitive processes, including communication, are disrupted and well-rehearsed, proceduralized skills are seemingly spared. All of these functions are relevant to police officer performance and decision-making ability during critical incidents directly have consequences to officer and public safety. Therefore, it is imperative to understand the effects of police officer physiological arousal (i.e., acute stress response) on tactical and communication skills during a critical incident. In the extant literature, few studies have considered these specific domains of police performance in the field, which hampers translation of the research to effective training interventions. A study that continually monitored police officer heart rates while on duty concluded that arousal was highest just prior to and during a critical incident, and officers did not fully recover before ending their shift (Anderson et al., 2002), which underscores the need for research simulations that enjoy high ecological validity. We aim to address this limitation in the current study by examining the relationship between acute physiological arousal and police officer tactical and communication skills during a simulated critical incident that was modeled after a real-life occurrence.

We evaluated police officer physiological arousal with complementary indicators taken from the cascade of biological responses following threat and stress. Cortisol is secreted from the pituitary-adrenocortical axis, and because it is one of the most critical hormones to increase short-term resilience to stress, it is a common target for field assessment of stress response (McEwen, 1998, 2006). Cortisol gradually increases following a stressor, reaching peak levels within 20–30 min and remains elevated for approximately an hour post-stress (Kirschbaum et al., 1993; Engert et al., 2011). Stress activates the sympathetic nervous system as evident by elevated heart rate (McEwen, 1998, 2006). Increased heart rate indicates greater arousal, and moderate rather than extreme increase and rapid recovery to baseline rate indicate an adaptive response (Schuler and O’Brien, 1997; McEwen, 1998, 2006). In addition, there are secondary effects of stress. For example, stress increases blood viscosity as an adaptive response to reduce the risk of fatal hemorrhage following a fight (von Känel et al., 2001). As a natural countermeasure, the anticoagulant, antithrombin, is released throughout the body’s vasculature, especially where large blood vessels bifurcate or blood flow is turbulent and there is increased risk of clotting (Arnetz and Ekman, 2006). Thus, increased antithrombin is an ideal response during a stressful encounter to minimize the risk of stress-induced blood clotting (von Känel et al., 2001; Arnetz and Ekman, 2006). A 5–10% elevation in antithrombin has been observed following stressful psychological tasks in a laboratory, with return to basal levels within 45 min post-stress (Austin et al., 2013). In the present study, we use change in cortisol and antithrombin from pre- to post-incident and maximum heart rate during the incident as indicators of individual physiological arousal.

With the use of these indicators of physiological arousal, we tested the hypothesis that greater stress response differentially affects police skills that rely on complex cognitive functions. Specifically, we hypothesize that greater increase in antithrombin and cortisol, as well as higher maximum heart rate, will predict lower scores on verbal and nonverbal communication with the suspect, which are conceived to have high cognitive demands. In contrast, elevation in physiological arousal indicators will predict better tactical skill, which is considered to rely predominantly on procedural memory.

## Materials and Methods

### Participants

Participants were 18 healthy, male police officers with 1 year of experience on the Swedish police force. Ages were not reported. All officers were fluent in Swedish and English. This sample has been described before in a previous report (Arnetz et al., 2009), and here, we report a novel investigation of the effect of physiological arousal on performance of specific skills (verbal and nonverbal communication and tactical skill). The sample reported here was selected randomly from a parent study of 75 police cadets who had participated in a behavioral intervention of imagery and skills training 1 year prior. A random sub-sample of cadets from the two conditions of the parent intervention study was invited to participate in the critical incident simulation. A total of 25 participants were invited to participate, based upon availability, facility in both English and Swedish language, and continued employment as police officers. Of the 25 invited, 18 cadets agreed to participate. At study completion, one individual had incomplete skill ratings and was removed from analysis; all analyses reported here include *N* = 17. The study was approved by the Karolinska Institute Ethics Committee, and all participating officers provided written informed consent.

### Critical Incident Simulation

The critical incident simulation was modeled after a real-life scenario that had resulted in the death of a police officer. The real-life incident was analyzed in detail, and the critical incident simulation was designed in consultation with police officers who were experts in police training. The critical incident simulation included multiple calls and potentially hostile encounters in order to elicit physiological arousal that is common to police officers.

The protocol for the incident was standardized across participants, and the critical incident and all associated physiological data collection were completed 8–10 am during the police training academy session. Each officer was equipped with a set of handcuffs, a loaded paintball gun, an extra round of ammunition, a closed radio system, and an unmarked police car. Officers were instructed to perform usual patrol work and were dispatched in pairs, consistent with typical protocol. Following deployment, the officers received three dispatch calls prior to the critical incident scenario. The first call requested assistance with an individual suspected of selling illicit drugs at the police academy building. Before arriving at the scene, the officer received a second call indicating that the suspect had moved to the shooting range where he was demonstrating aggressive and hostile behavior toward the public. Officers pursued the suspect to the shooting range, and before arriving, they received a third call that redirected the officer to investigate a suspicious vehicle at a restricted military airfield. Before completing the call, the officer was called off again and redirected to investigate the critical incident – the scene of a post office robbery. Police officers were directed to the critical incident approximately 90 min after deployment.

Dispatch informed the officer that a post office had been robbed, the suspect had fled the scene, and the identity of the informant who had reported the incident was unknown. The officer was instructed to meet the building maintenance manager inside an exterior door, which had been damaged during the robbery. These instructions were designed to lower the police officer expectations of the degree of danger in the call, creating a surprising hostile encounter.

Upon arrival at the scene, the police officer observed the informant (an actor) standing 60 yards away from the building in an open field, and the informant was pointing at the damaged exterior door. The officer then walked 15–20 yards to approach the door. Without warning, two masked and heavily armed suspects (actors) exited the building. The suspects took aim at the police officers, and the first suspect immediately fired a shot from his paintball weapon. The second suspect turned right and stopped. The second suspect only spoke English, and although participating officers were fluent in English, they were not instructed of this requirement prior to the incident. The actors portraying the suspects were instructed to follow the police officers’ clear, unambiguous orders when they reasonably comprehended the meaning (i.e., in a language they understood or communicated *via* body language and hand signals). The simulation ended when the police officer handcuffed the suspects. The critical incident was completed within approximately 30 min (within 2 h of deployment in total).

### Police Skill Assessment

Police officers performed the critical incident simulation in pairs, but performance was rated individually. An independent police officer, who was an expert in the subject matter, observed the critical incident simulation from a rooftop vantage point. Each police officer was rated in eight domains: tactics, verbal communication, nonverbal communication, material management/dexterity, self-control, control of the suspect, control of the public, and confidence in incident management safety. Each domain was rated on a scale ranging from “poor” (0) to “excellent” (100). Scores across the domains were summed to calculate Total Performance Rating, which ranged from 0 to 800, and higher scores indicated better overall performance (Arnetz et al., 2009). Total Performance Rating is a summary measure that is comparable to a job performance rating a police officer may receive in the academy or on duty. Here, the measure was used to assess effects on general performance in a preliminary analysis. The hypothesized differential effects of physiological arousal were tested with specific scale ratings for verbal and nonverbal communication and tactical skill.

### Physiological Arousal Indicators

On the day of the critical incident simulation, the police officers fasted in the morning. Blood samples were collected prior to the critical incident simulation, following which the officers ate a standard breakfast meal before deployment to the simulation scenario. A second blood sample was collected immediately following simulation completion, with no more than a 15-min delay. Circulating antithrombin and cortisol in blood serum were measured using standard laboratory tests. Increase in each of these biomarkers is consistent with a healthy, adaptive response to stressful situations (McEwen, 1998, 2006; von Känel et al., 2001). To measure change in circulating antithrombin and cortisol, the difference of post-incident measure from pre-incident measure was calculated, and positive change scores indicated an increase. Heart rate was used as an indicator of psychophysiological arousal. Officers wore a portable monitor to measure heart rate before and after the simulation, as well as continuously throughout the incident. The baseline heart rate measure was collected during the simulation before the dispatch call for the critical incident, and final heart rate measurements were taken within 10 min of simulation’s conclusion. Because heart rate was expected to recover quickly in this sample of healthy cadets, maximum heart rate during the incident was used as a proxy indicator of maximum arousal during the critical incident. In secondary analyses, change in heart rate from pre- to post-incident was also tested as a predictor of skill rating.

### Statistical Analysis

All statistical analysis was completed in SPSS (v.23) software. Prior to hypothesis testing, change scores for cortisol and antithrombin were calculated, and all data were screened for normality and univariate outliers. Preliminary analysis included paired t-tests of change in heart rate, antithrombin and cortisol from pre- to post-incident, and a linear regression to assess a possible general effect of these physiological arousal indicators on Total Performance Rating. Following this preliminary analysis that provides important contextual information, the hypothesis was tested in a repeated measures general linear model (GLM) that included a three-level skill factor (tactical skill, verbal communication, and nonverbal communication) as a dependent variable predicted by the physiological indicators. The three physiological arousal indicators – maximum heart rate during the critical incident, change in cortisol, and change in antithrombin – were weakly correlated (*r* = −0.27 to 0.04, all *p* ≥ 0.29), and therefore, all indicators were entered simultaneously to predict differences in skill rating. Significant omnibus *F*-tests of the interaction term skill × physiological indicator were decomposed by Pearson bivariate correlations. Significance testing was set as *p* < 0.05 for all tests. In secondary analysis with the same repeated measures GLM structure, we assessed change in heart rate as an alternate to maximum heart rate predicting skill rating. Effect size estimates are reported for each test coefficient: within-subjects Cohen’s d for estimates from paired t-tests and partial eta squared ($\eta_{p}^{2}$) for GLM linear regression coefficients. The current sample provided sufficient sensitivity to find at least a moderate effect size of change in arousal indicators (*d* = 0.63), a moderate-large size arousal indicators predicting performance skill (*f*^2^ = 0.87), and difference between skills (*f* = 0.40) to significance (all estimates assuming power = 0.80, *α* = 0.05; Faul et al., 2009). Based upon the available sample size and informed by the literature, we have conservatively selected hypothesis tests that would be sufficiently powered.

## Results

### Physiological Arousal Indicators

Prior to hypothesis testing, we examined the distributions in physiological indicators of stress and possible mean changes in these measures between pre- and post-critical incident simulations. Heart rate (beats/min) significantly increased from pre- (*M* = 67.94, *SD* = 8.74) to post-encounter [*M* = 83.47, *SD* = 11.06; *t*(16) = −4.77, *p* < 0.001, *d* = 1.16], and during the encounter, maximum heart rate was substantially elevated [*M* = 157.82, *SD* = 15.59; *t*(16) = −18.54, *p* < 0.0001, *d* = 4.50]. As compared to before the encounter, antithrombin (AT kIE/L) demonstrated a nonsignificant trend for increase [*t*(16) = −2.12, *p* = 0.05, *d* = 0.51] and cortisol (nmol/L) demonstrated a nonsignificant trend for decrease [*t*(16) = 2.05, *p* = 0.06, *d* = 0.50]. Although tests of mean change in circulating markers failed to reach statistical significance, individuals varied in the magnitude of change in antithrombin (*M* = 0.06, *SD* = 0.12) and cortisol (*M* = −81.29, *SD* = 163.62), as well as change in heart rate (*M* = 15.53, *SD* = 13.43), and we hypothesized that individual differences in these physiological markers will predict differences in performance. Because heart rate was expected to recover quickly, maximum heart rate during the encounter was used as a proxy for maximum stress response in the moment and change in antithrombin and change in cortisol as additional markers of the cumulative physiological response. Secondary analyses further tested change in heart rate as a predictor of skill rating.

### Mixed Effects of Physiological Arousal on Police Skills

**Figure 1:**
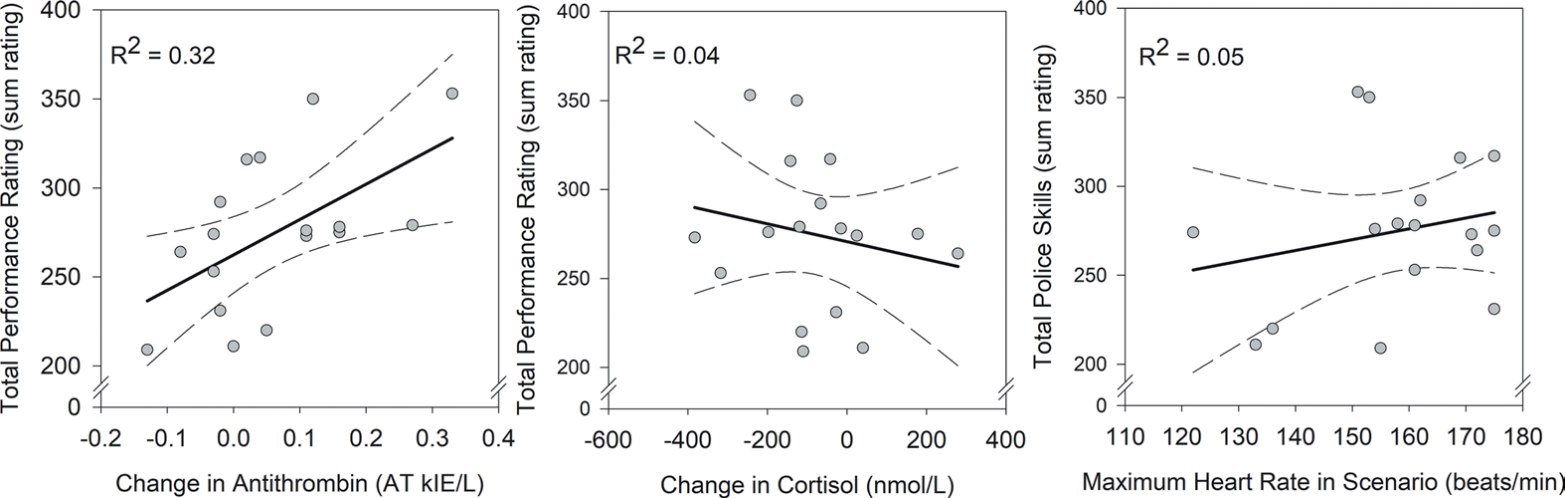
Correlations between indicators of physiological arousal and Total Performance Rating. The regression model that included all three indicators of physiological arousal identified change in antithrombin as a significant predictor [*F*(1, 13) = 5.46, *p* = 0.036, $\eta_{p}^{2}$ = 0.30], whereas change in cortisol [*F*(1, 13) = 0.05, *p* = 0.83, $\eta_{p}^{2}$ = 0.004] and maximum heart rate during the encounter [*F*(1, 13) = 0.84, *p* = 0.38, $\eta_{p}^{2}$ = 0.06] were unrelated to overall performance. The correlations are depicted with bolded, solid lines and 95% confidence intervals are displayed with broken lines; bivariate coefficients are reported for each.

During the simulated encounter, an expert officer rated the performance of each participating police officer across multiple domains, and Total Performance Rating was used as an index of overall performance in preliminary analysis. Total Performance Rating scores ranged from 209 to 353. Greater increase in antithrombin over the course of the simulated encounter was associated with greater overall performance: *F*(1, 13) = 5.46, *p* = 0.036, $\eta_{p}^{2}$ = 0.30 (Figure 1). Change in cortisol [*F*(1, 13) = 0.05, *p* = 0.83, $\eta_{p}^{2}$ = 0.004] and maximum heart rate during the encounter [*F*(1, 13) = 0.84, *p* = 0.38, $\eta_{p}^{2}$ = 0.06] were unrelated to overall performance (Figure 1). The model including the three physiological indicators of stress explained approximately 36% of variance in performance (*R*^2^ = 0.359). This analysis provides important contextual information to interpret the hypothesis test.

**Figure 2:**
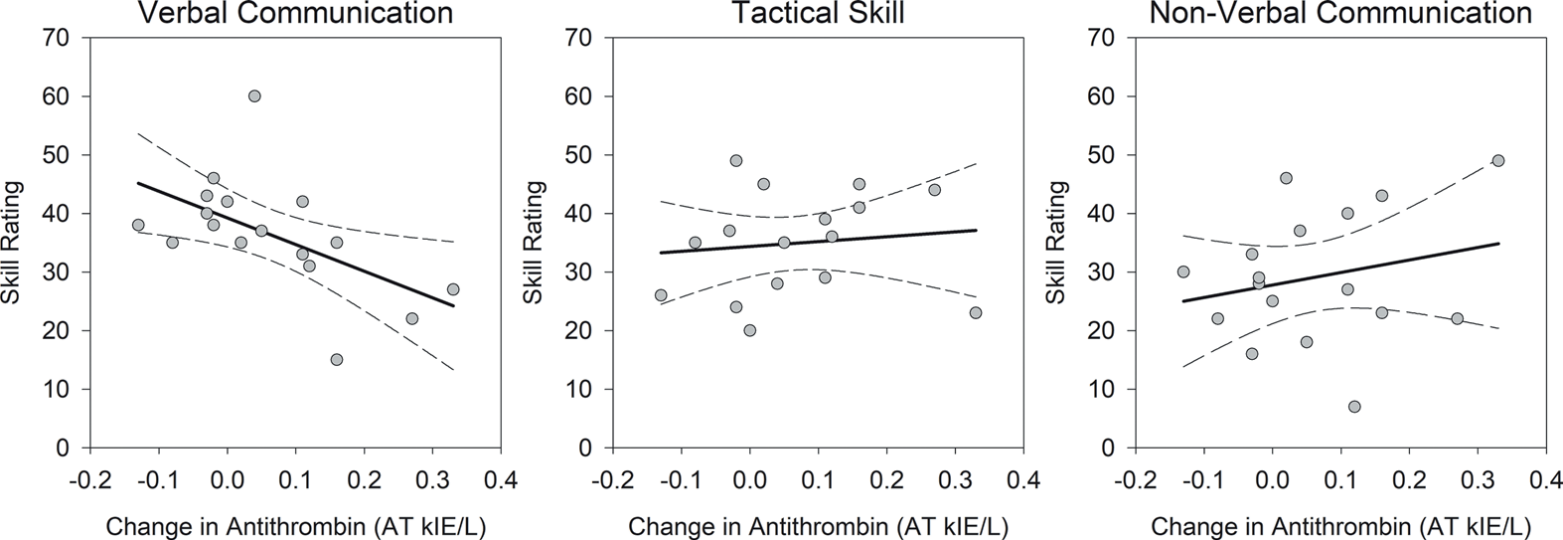
Differences in police officer skill performance predicted by change in antithrombin. Change in antithrombin differentially affected the examined police skills: *F*(2, 12) = 4.08, *p* = 0.04, $\eta_{p}^{2}$ = 0.41. Greater increase in antithrombin during the simulated critical incident was associated with worse verbal communication (*r* = −0.56, *p* = 0.02). The correlations with tactical skill (*r* = 0.12, *p* = 0.66) and nonverbal communication (*r* = 0.23, *p* = 0.37) were not significant, but positive. The correlations are depicted with bolded, solid lines and 95% confidence intervals are displayed with broken lines.

The hypothesis that the effects of physiological arousal may differentially affect specific skills was tested in a repeated measures GLM. The range of scores for tactical skill (range = 20–49; *M* = 34.88), verbal communication (range = 15–60; *M* = 36.41), and nonverbal communication (range = 7–49; *M* = 29.12) were comparable, and mean ratings did not significantly differ between skills [*F*(2, 12) = 1.91, *p* = 0.19, $\eta_{p}^{2}$ = 0.24]. The effects of greater increase in antithrombin were differential across police skills [Skill × Change in Antithrombin, *F*(2, 12) = 4.08, *p* = 0.04, $\eta_{p}^{2}$ = 0.41]. Greater increase in antithrombin was associated with worse verbal communication (*r* = −0.56, *p* = 0.02), explaining approximately 32% of variability in performance (Figure 2). Although not significant, increase in antithrombin was associated with better tactical performance (*r* = 0.12, *p* = 0.66) and nonverbal communication (*r* = 0.23, *p* = 0.37). Change in cortisol [*F*(2, 12) = 0.38, *p* = 0.69, $\eta_{p}^{2}$ = 0.06] and maximum heart rate [*F*(2, 12) = 1.26, *p* = 0.32, $\eta_{p}^{2}$ = 0.17] did not differentially predict skill ratings. Taken together, greater increase in antithrombin was associated with overall better police performance during a critical incident, but a specific deficit in verbal communication.

In a secondary analysis, change in heart rate was considered as an alternate to maximum heart rate to predict individual differences in performance. Change in heart rate did not predict differences in Total Performance Rating [*F*(1,13) = 0.01, *p* = 0.91, $\eta_{p}^{2}$ = 0.001]. Greater increase in heart rate differentially effected skill ratings [Skill × Change in Heart Rate, *F*(2, 12) = 4.08, *p* = 0.04, $\eta_{p}^{2}$ = 0.41]. Examining Pearson correlations, no single correlation was statistically significant but the direction of effects differed: greater increase in heart rate was negatively correlated with verbal communication (*r* = −0.35, *p* = 0.17) and positively correlated with tactical skill (*r* = 0.11, *p* = 0.69) and nonverbal communication (*r* = 0.26, *p* = 0.32). Accounting for the effects of change in heart rate, change in antithrombin remained a significant predictor in the model [*F*(1, 12) = 5.28, *p* = 0.02, $\eta_{p}^{2}$ = 0.47]. In summary, greater increase in antithrombin and greater increase in heart rate – indicators of greater physiological arousal – were associated with lower verbal communication skill, whereas effects on nonverbal communication and tactical skills were not significant and trended in the positive direction.

## Discussion

The present study provides a unique view of the physiological predictors of police officer performance during stressful encounters. The realistic critical incident simulation provided a strong analogue to real-world performance. The officers were forced to respond to a rapidly changing environment, shifting objectives, and uncertainty regarding potential threats. The simulated critical incident appears to have induced stress similar to a real-life experience. As evidence of the simulation’s efficacy, police officers demonstrated increased heart rate during the scenario and trends for elevated antithrombin. The result was an ecologically valid and rich assessment of police officer performance across multiple domains: tactics, verbal communication, and nonverbal communication, for which skill ratings were differentially predicted by physiological arousal.

In the present sample, elevated antithrombin emerged as a significant predictor of police officer performance. Antithrombin plays a central role in countering the body’s chemical cascade mechanisms that are involved in creating blood clots during trauma; thus, increased antithrombin has been considered an adaptive anticoagulant response that is desirable during stress (Yanada et al., 2002). Observed elevation in antithrombin and other anticoagulants persists for an extended time after the stressor has been removed, presumably for its benefits to reduce the risk of blood clot from trauma that may be favored in evolution (Austin et al., 2013). As an index of physiological arousal, greater increase in antithrombin predicted higher Total Performance Rating. Total Performance Rating (the sum across multiple skill ratings) is comparable to the assessments police officers would receive in training and when on duty and represents a summary of job performance across multiple skill domains. The result is consistent with the expectation of healthier physiological responses predicting better performance in the field. Several laboratory studies have demonstrated that moderate levels of physiological arousal are adaptive and positively correlate with skill acquisition, accuracy, and long-term retention (Yerkes and Dodson, 1908; Sandi, 2013). The Total Performance Rating reported here includes multiple types of skills, some that are complex and explicit (i.e., verbal communication), some that are conceptually related to proceduralized or implicit skill (i.e., tactical skill), as well as less complex skills (i.e., materials management) and self-referential ability (i.e., self-control, confidence). In this manner, the Total Performance Rating provides a nonspecific summary of job performance, and it is in this measure that we identified a positive correlation between physiological arousal and performance.

Yet, when specific police skills were considered distinctly – verbal and nonverbal communication that are complex skills and tactical skill that included well-rehearsed behaviors – increased antithrombin was not universally positive. Specifically, greater increase in antithrombin was negatively associated with verbal communication, and a nonsignificant, but positive, association with tactical skill and nonverbal communication. Our hypothesis was partially supported, finding evidence of deficits in verbal communication, although the effects on nonverbal communication and tactical skill were not statistically significant.

Considering elevated antithrombin as an indicator of the police officer stress response, it is plausible that skills that were more effortful were differentially affected. This is consistent with another study that reported high acute stress and high trait anxiety negatively correlated with verbal communication by police officers during an arrest (Renden et al., 2017a). Verbal communication is cognitively demanding, requiring attentional and processing resources that, during a critical incident, would be also in use by visual scanning and working memory as the officer assessed the scene. When under psychological and emotional stress, effortful and explicit cognitive functions, like those required for verbal communication, tend to falter (Lupien et al., 1999; Newcomer et al., 1999; Oei et al., 2006; Sandi, 2013). Stress-related impairments to declarative memory (Morgan et al., 2004; Taverniers et al., 2013), which we speculate may have contributed to poor verbal communication in this study, is consistent with the evidence of inaccurate officer reporting in post-incident reports and debriefing that is associated with stress response and fatigue (see Hope, 2016 for a review). In this study, the cognitive demands for verbal communication were likely heightened because the Swedish officers were required to speak to the suspects in a language they were fluent in, but not native to. To our knowledge, the effects of acute or chronic stress on verbal communication in police officers during a critical incident have not been well studied. As communication is a critical strategy in resolving an incident without lethal force, further research in this area may identify targets for training intervention.

In contrast to the negative effect on verbal communication, nonverbal communication and tactical response were weakly, but positively, correlated with increased antithrombin. It is possible that both skills relied more upon procedural knowledge and thereby were more resilient to physiological arousal in the stressful encounter (Kirschbaum et al., 1996; Lupien et al., 1997; Sandi, 2013). Officers can train for a variety of scenarios, emphasizing the use of core tactics that will be employed in a multitude of situations (e.g., how to hold a firearm, the techniques for subduing a subject to apply handcuffs). These specific techniques can be drilled and repeated to the point of automaticity, thereby reinforcing procedural memory. Similarly, common nonverbal cues can be rehearsed and become like habits. Bearing this interpretation, prior intervention studies that have used repeated rehearsal have found that behavioral skills, such as tactical self-defense, are resilient to the effects of acute stress (Shipley and Baranski, 2002; Renden et al., 2015, 2017b; Andersen and Gustafsberg, 2016). However, other observational studies report a negative effect of acute stress on tactical behaviors, such as hand-cuffing and arrest procedures (Renden et al., 2014) and the use of force while controlling a suspect (Hope, 2016). Considering the available evidence, it is plausible that with intentional training, rehearsed tactical skills and nonverbal communication may be resilient to acute stress during a critical incident. Although the details of a situation will change, these well-rehearsed techniques can be relied upon during a stressful event, unlike verbal communication that cannot be easily stereotyped.

Effective communication during a police encounter will necessarily depend upon the utterances of the individuals involved. Determining basic verbal commands that can be rehearsed to aid automaticity, even during stressful encounters, may be a useful target for future cadet training programs. However, proceduralized skills by nature are inflexible and there may be a risk of an officer relying too much on such skills when under duress. Much as tunnel vision may unduly restrict situational awareness, an over-reliance on proceduralized communication skills may interfere with the breadth of verbal engagement that is sometimes required in a critical incident. As an alternative, police officers could instead rehearse techniques to increase flexibility and awareness during stressful situations, thereby allowing for the cognitive freedom to engage in improved verbal performance (Arble et al., 2017).

If we extend this evidence to police officer decision-making and performance in the real world, there are two important implications. First, successful decision-making among police officers relies upon several distinct skill domains, across which the officer may be differentially successful. Returning to the previous example of approaching a potentially armed suspect, an officer may be quite successful in positioning himself/herself as to avoid attack and may also be successful in identifying the presence of a weapon. However, failure to verbally engage with the suspect and deescalate the situation could undo the successes achieved in the other domains. Second, articulating an ideal physiological response for police officers is quite complex. The police officer health, personal history, and baseline anxiety may modify their ideal physiological responsiveness (Otte et al., 2005; Daus and Brown, 2012). Furthermore, the psychophysiological needs for quick reaction times and visual scanning may not be entirely congruent with the needs required to address an individual in a calm, authoritative manner. Experiments and interventions focusing on one performance domain (e.g., decisions to shoot) as a measure of decision-making may wish to consider the addition of alternative performance domains to address this complexity.

The present results identify the importance of physiological responsiveness as a predictor of police officer performance in response to critical incidents. However, not all physiological indicators of stress were associated with performance – we did not find evidence of maximum heart rate during the scenario or change in cortisol correlating with performance. A constrained elevation in heart rate is ideal for performance (McEwen, 2002) and is of particular importance during critical decision-making moments within an encounter (Arnetz et al., 2009). In this sample, heart rate was elevated to indicate arousal in the scenario but did not predict individual differences in police skills. Similarly, the release of cortisol is theorized to be beneficial during dangerous situations, providing an increase in vigilance and arousal (Otte et al., 2005; McEwen, 2006). It is further believed that an elevation in cortisol levels during stressful experiences will subsequently assist in returning the body to a state of homeostasis, thereby facilitating successful recovery (McEwen, 1998, 2006).

Following this evidence, we expected a moderate increase in cortisol during the training scenario to predict better performance, but here we found a nonsignificant trend for decrease in this sample. The statistical trend indicating decrease in cortisol may be an artifact of measures that were taken in the morning (8–10 am), as cortisol is naturally high in the early circadian rhythm and decreases with waking time (Hellhammer et al., 2009). The failure of cortisol to emerge as a significant predictor of performance may also reflect the complex nature of the hormone and its effects. Excessive cortisol activation has been associated with negative outcomes, both physical (Whitworth et al., 2005) and emotional (Carroll et al., 2007). Furthermore, there is evidence to suggest that the relationship between cortisol and cognitive function may be moderated by several variables, including previous trauma (Yehuda, 2002). In the present context, the role of anticipatory anxiety may be particularly important. When inducing cortisol *via* anticipatory stress, studies suggest that individuals become riskier in their decision-making and less accurate overall (Starcke et al., 2008). Thus, although cortisol activation may prove adaptive for some, for others (particularly those with high anticipatory anxiety), the hormone may prove a hindrance. The current study design precludes tests of possible moderators of the effects of cortisol or heart rate, and therefore, we cannot presently test this hypothesis. Future studies of larger samples that include detailed personal and health histories would be positioned to evaluate the importance of individual factors in police officer decision-making under stress.

The reported evidence should be interpreted with consideration of its limitations. First, the analyses are reported from a small sample of cadets, which was sufficient to detect some hypothesized effects to significance, but tests of cortisol and heart rate may be underpowered. Further, we were unable to effectively test possible nonlinear relationships between physiological markers and performance ratings in a sample of this size nor was the sample sufficient to power a larger model testing differential effects across all of the performance domains that were rated during the simulation. This warrants a larger follow-up study to address these important research questions. Second, the sample was selected to include male cadets and does not represent the diversity of the international community of police officers. Nonetheless, we report on physiological markers of stress that are well validated and typical police skill domains, and therefore, the results here can be applied to understanding the effects of physiological arousal and stress on police performance across communities. Physiological response to acute stress (Novais et al., 2016) and associated behavioral consequences (Allwood and Salo, 2012; Arble et al., 2018) varies as a function of age and sex, which we cannot consider here due to the selection of only male cadets and age was not reported in the study. Third, the analyses are correlational and based upon observational data and therefore cannot comment on causality. The physiological response to psychological and emotional stress is well established, and we take advantage of this knowledge in this study design, but we do not directly manipulate circulating biomarkers. Fourth, the sample was drawn from a parent study that included a behavioral intervention of imagery and skills training 1 year prior. The sample in the present report represents both the control (50%) and intervention conditions (50%), selected at random. We do not test possible intervention-related differences in the present analysis, which were not hypothesized, and the small sample size is expected to be insufficient for such a test. The degree to which intervention may modify the differential effects of stress on communication is left to future studies to address. Finally, the simulation was designed as an analogue to an actual event and was developed to be conducted within a police academy training setting. This setting, though adding some ecological validity, provided logistical constraints. For example, all testing was done in the morning hours and possible time-of-day effects that are relevant for circulating cortisol could not be examined. Further, under different simulation parameters, with a team of multiple raters with complementary areas of expertise (e.g., an expert in police tactics in addition to an expert in communication) could have provided a more nuanced evaluation of police officer performance.

## Conclusion

The present study demonstrates that physiological arousal during a critical incident differentially affects police officer verbal communication, nonverbal communication, and tactical skills. These differential effects speak to the complex nature of effective police work, the difficulties in assessing and defining ideal police performance, and further suggest that police training must address the array of intrapersonal and situational demands facing officers in the field. Future studies may consider adaptive training interventions that leverage nonverbal communication and tactical skills that appear to be robust to the effects of physiological arousal during a critical incident.

## Ethics Statement

The study was approved by the Karolinska Institute’s Ethics Committee and all participating officers provided written informed consent.

## Author Contributions

EA and AD conducted data analysis, data interpretation, and prepared the manuscript. BA conceived the study, conducted the study, was awarded funding for the study, and prepared the manuscript.

### Conflict of Interest Statement

The authors declare that the research was conducted in the absence of any commercial or financial relationships that could be construed as a potential conflict of interest.
